# Clinical and Hematological Characteristics of Vitamin B12 Deficiency and Evaluation of the Therapeutic Response to Vitamin B12 Supplementation

**DOI:** 10.7759/cureus.76468

**Published:** 2024-12-27

**Authors:** Aditya R Agrawal, Navin Mair, Rishi S Mehta, Arjun S Chakrapani, Kajal Gupta, Yashraj Srivastav, Gaurav Mittal

**Affiliations:** 1 Otolaryngology, K. J. Somaiya Hospital and Research Centre, Mumbai, IND; 2 Nephrology, Sir H. N. Reliance Foundation Hospital and Research Centre, Mumbai, IND; 3 Surgery, Mahatma Gandhi Mission Hospital, Navi Mumbai, IND; 4 Orthopaedics, Apollo Speciality Hospitals, Perungudi, Chennai, IND; 5 General Medicine, Dr. D. Y. Patil Medical College, Hospital & Research Centre, Navi Mumbai, IND; 6 Emergency Medicine, Rajiv Gandhi Institute of Medical Sciences, Adilabad, IND; 7 Research and Development, MediBrains Social Welfare Foundation, Mumbai, IND; 8 Internal Medicine, Mahatma Gandhi Institute of Medical Sciences, Wardha, IND

**Keywords:** cobalamin, hematological changes, macrocytic anemia, neurological symptoms, supplementation, vitamin b12 deficiency

## Abstract

Background

Vitamin B12 deficiency, or cobalamin deficiency, is common among populations with low consumption of animal-based products, mainly in India, due to religious and socioeconomic factors, which significantly increase the deficiency rate. The condition has been characterized by a wide range of clinical and hematological symptoms, mainly affecting the blood and nervous system. This study aims to assess the clinical and hematological characteristics of patients with vitamin B12 deficiency and assess the therapeutic response to supplementation with vitamin B12.

Methodology

This two-year, in-hospital study was conducted at K. J. Somaiya Medical College and Hospital. The study involved 180 patients aged between 18 and 70 years with hemoglobin below 13 g/dL for males, less than 12 g/dL for females, and serum vitamin B12 levels below 250 pg/mL. Data on demographic and clinical characteristics of the patients, along with hematological parameters, including complete blood count, peripheral smear, reticulocyte count, and bone marrow examination, were collected. Each patient received six intramuscular injections per week of 1,000 µg of vitamin B12. The hematological parameters were measured at different follow-up intervals, and the therapeutic response was measured using the one-way analysis of variance. We employed Pearson’s correlation coefficient to investigate the relationship between the severity of anemia and vitamin B12 level.

Results

The study showed a higher prevalence of male patients, accounting for 105 (58.3%) of the sample, with the majority of patients aged between 46 and 60 years, totaling 70 (38.9%). Common comorbidities included hypertension in 60 (33.3%) and diabetes in 45 (25%) patients. The most frequently reported symptom was fatigue, present in 120 (66.7%) patients, followed by neurological symptoms such as tingling and numbness in the extremities reported by 98 (54.4%) patients. Baseline hematological assessments indicated macrocytic anemia, with a mean hemoglobin level of 9.7 g/dL and an elevated mean corpuscular volume (MCV) of 104.7 fL. At the end of six weeks of vitamin B12 therapy, there were notable improvements, with hemoglobin levels rising to 12.6 g/dL, MCV decreasing to 91.3 fL, and reticulocyte count increasing to 2.1%. A strong positive correlation was observed between hemoglobin levels and serum vitamin B12 concentrations (r = 0.75, p < 0.001).

Conclusions

Vitamin B12 deficiency leads to clinical and hematological abnormalities, including macrocytic anemia and neurological symptoms. This study demonstrates that vitamin B12 supplementation effectively reverses these abnormalities, improving both hematological and neurological outcomes. Given the prevalence of deficiency, routine screening and supplementation are recommended, particularly for at-risk populations such as vegetarians and older adults. Further research is needed to assess the long-term effects of supplementation, particularly its potential role in reducing cardiovascular risk in individuals with vitamin B12 deficiency.

## Introduction

A deficiency in vitamin B12, or cobalamin insufficiency, refers to a condition where blood levels of vitamin B12 are lower than normal. Animal-based foods such as meat and eggs serve as major sources of cobalamin. The common Indian diet frequently lacks sufficient levels of cobalamin due to a combination of religious practices and economic factors, making this deficiency widespread in the population [1,2]. Reports indicate that 47-71% of Indian adults are affected by this deficiency [3,4]. Vitamin B12 deficiency manifests through various symptoms that develop gradually and can worsen without proper treatment. In its early stages, the deficiency may go unnoticed, but as it progresses, symptoms such as fatigue, dizziness, rapid heart rate, fast breathing, and pale skin may occur due to anemia. Other symptoms can include simple bruising or bleeding, such as from the gums, digestive issues, and disturbances, such as a sore tongue, stomach upset, weight loss, and either diarrhea or constipation.

If left untreated, vitamin B12 deficiency can cause nerve damage, leading to tingling or numbness in the extremities, difficulty walking, emotional changes, depression, cognitive difficulties, disorientation, and, in severe instances, dementia. Additionally, the deficiency is linked to elevated homocysteine levels in the blood, a known risk factor for cardiovascular diseases [5]. Hematological changes associated with vitamin B12 deficiency can present with blood results that appear normal to the typical signs of megaloblastic anemia. These include increased mean corpuscular volume (MCV), macro-ovalocytes, hypersegmented neutrophils (having six or more lobes) on peripheral blood smears, reduced reticulocyte count, and a modest rise in indirect bilirubin levels. While red blood cell levels are generally reduced in cases of mild-to-moderate anemia, and white blood cells and platelet counts may also be diminished significantly in more severe instances.

In the bone marrow, distinctive abnormalities are observed, such as marked erythroid hyperplasia due to impaired generation of red blood cells. In the erythroid lineage, megaloblastic alterations are evident, marked by increased cell dimensions and a mismatch in the nucleus and cytoplasm, where cytoplasmic growth progresses as compromised DNA synthesis hinders the maturation of the nucleus. In the myeloid lineage, large band forms and metamyelocytes are frequently observed. Thus, megaloblastic anemia must be considered a crucial differential diagnosis for individuals with pancytopenia [6,7].

Diagnosis of vitamin B12 deficiency is primarily confirmed by assessing the concentration of the vitamin in the bloodstream, with increased levels of methylmalonic acid serving as additional indicators of a deficiency. The management approach consists of administering vitamin B12 through oral or injectable forms; initially, higher daily doses are provided, which are then reduced to less frequent lower doses as improvement occurs. If an underlying reversible cause is identified, it should be addressed, but in cases where no such cause is found, lifelong supplementation may be required. Effective treatment typically reverses megaloblastic changes within 24 hours and restores normal bone marrow function in 48 hours. Red blood cell counts and hemoglobin levels improve within one week, while the complete normalization of blood counts may take around six to eight weeks [8].

This study aims to assess the clinical and hematological characteristics associated with vitamin B12 deficiency and evaluate the therapeutic response to vitamin B12 supplementation in affected patients. This includes identifying the range of symptoms, hematological changes, and the effectiveness of treatment in restoring normal blood parameters and resolving clinical symptoms.

## Materials and methods

Study design and setting

This longitudinal study was conducted in a hospital setting at K. J. Somaiya Medical College and Hospital over a two-year period from March 2022 to March 2024. Ethical approval for the study was obtained from the Institutional Ethics Committee, and informed written consent was obtained from all participants before their inclusion. Patients admitted to the medicine ward with hemoglobin counts of less than 13 g/dL in males and less than 12 g/dL in females, indicating anemia, were evaluated for vitamin B12 deficiency. Serum vitamin B12 estimation was performed using a chemiluminescent immunoassay. In total, 180 patients with serum vitamin B12 values of less than 250 pg/mL were selected.

Sample Size Calculation

The sample size of 180 patients was determined through a power analysis to ensure adequate statistical power to detect meaningful differences and associations within the study variables. The target power was set at 80%, which is commonly used in clinical research to minimize Type II errors. A moderate effect size was assumed based on prior studies on vitamin B12 supplementation, with a significance level of 0.05. This sample size allowed for sufficient representation of demographic variables, comorbidities, and clinical symptoms while accounting for potential attrition. It ensured greater precision in estimating treatment effects, more reliable statistical results, and the ability to conduct subgroup analyses. This sample size was deemed adequate to achieve the research objectives, improving the validity of the findings related to the impact of vitamin B12 supplementation on hematological parameters and clinical symptoms.

Selection Criteria

Patients included in the study were diagnosed with anemia, defined as hemoglobin levels below 13 g/dL for males and below 12 g/dL for females, along with serum vitamin B12 levels below 250 pg/mL. While plasma vitamin B12 levels below 200 pg/mL are commonly used as the diagnostic threshold for deficiency, we adopted a higher cutoff of 250 pg/mL to include patients at the lower end of the reference range who exhibited clinical and hematological features consistent with vitamin B12 deficiency, such as macrocytosis indicated by increased MCV. This approach aimed to capture a broader spectrum of patients, including those with early-stage deficiency, for a comprehensive evaluation of clinical and therapeutic outcomes.

Patients with comorbid conditions, such as hypertension and diabetes, were intentionally included in the study, as these conditions frequently coexist with anemia and may influence its clinical and hematological presentation. Including patients with comorbidities allowed the study to reflect a real-world population commonly encountered in clinical practice, enhancing the applicability of the findings. Informed consent was obtained from all participants before their enrollment, ensuring ethical compliance and clarity regarding the study procedures.

Data sources and variables

Comprehensive data collection was performed for all participants, encompassing demographic details, presenting complaints, and findings from general and systemic examinations. A standardized proforma was used to systematically record clinical and laboratory data. Laboratory investigations included a complete blood count (CBC), peripheral blood smear, reticulocyte count, and, where necessary, bone marrow examination conducted with informed consent. Additional tests included liver function tests (LFTs), renal function tests (RFTs), and serum vitamin B12 levels for a detailed assessment of the patients’ hematological and biochemical status.

The treatment regimen consisted of six intramuscular (IM) injections of 1,000 µg vitamin B12 administered weekly. The decision to use high-dose IM supplementation was based on clinical guidelines and the need for rapid restoration of vitamin B12 levels, particularly in patients with severe deficiency or malabsorption issues. IM administration ensures efficient absorption by bypassing the gastrointestinal tract, providing a more reliable therapeutic response. Participants were informed about the potential, though rare, side effects, such as nausea, headaches, erythema, chromaturia, and local injection-site reactions.

Patients were closely monitored throughout the study period, with follow-up assessments conducted at the end of weeks one, three, and six. During each follow-up, repeat CBC and peripheral blood smear analyses were performed to evaluate hematological improvements. The primary outcomes included clinical symptom resolution and significant changes in hematological parameters, such as increases in hemoglobin levels, normalization of MCV, and elevation of reticulocyte count, reflecting the efficacy of vitamin B12 supplementation.

Statistical analysis

Continuous data were reported as mean values and standard deviations, whereas categorical data were represented as frequencies and percentages. Hematological parameters were analyzed at various follow-up intervals and were compared using the one-way analysis of variance (ANOVA) test. Pearson’s correlation coefficient was used to assess the relationship between the severity of anemia and serum vitamin B12 levels. A p-value of less than 0.05 was considered statistically significant. Data analysis was performed using SPSS software version 23.0 (IBM Corp., Armonk, NY, USA).

## Results

**Figure 1:**
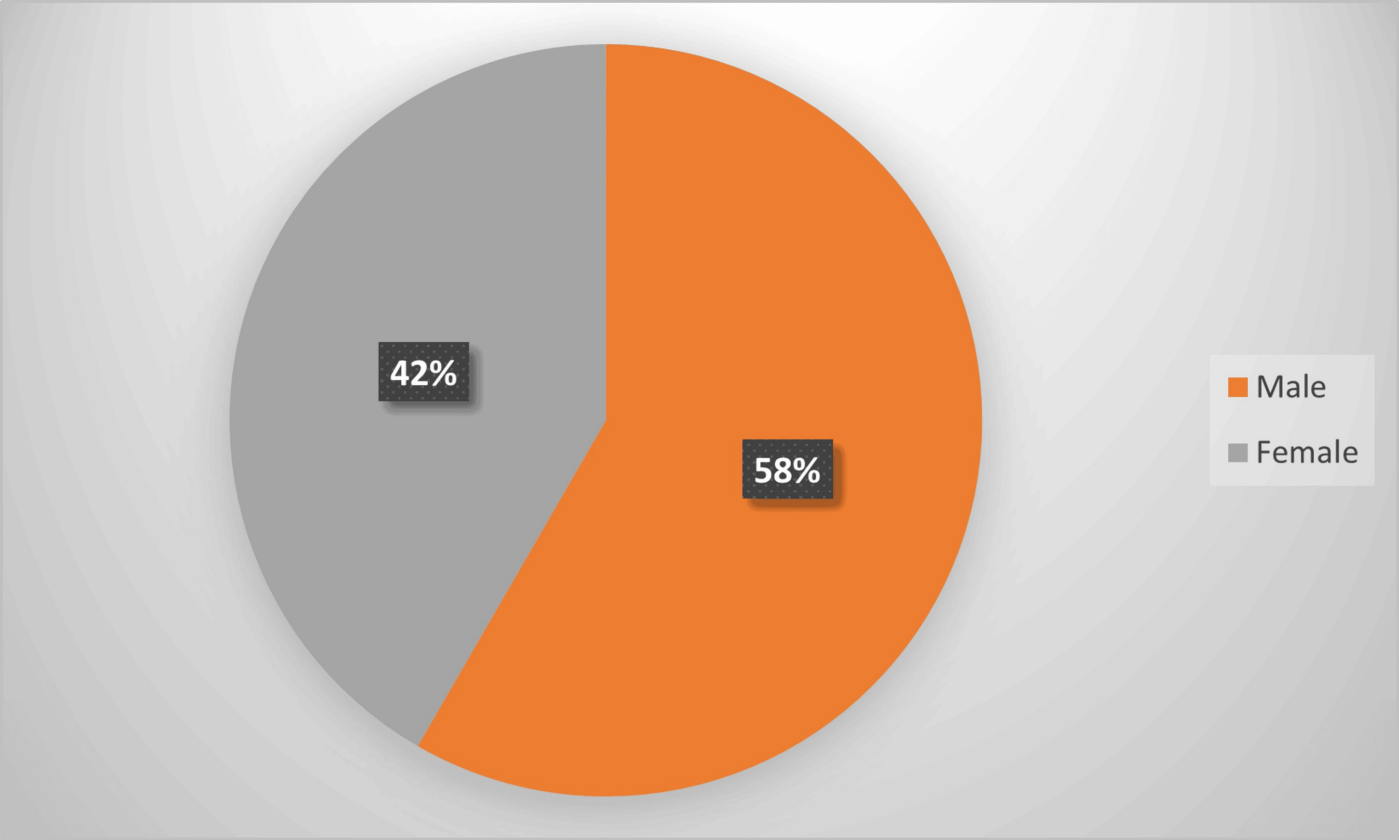
Gender distribution of the study participants.

**Figure 2:**
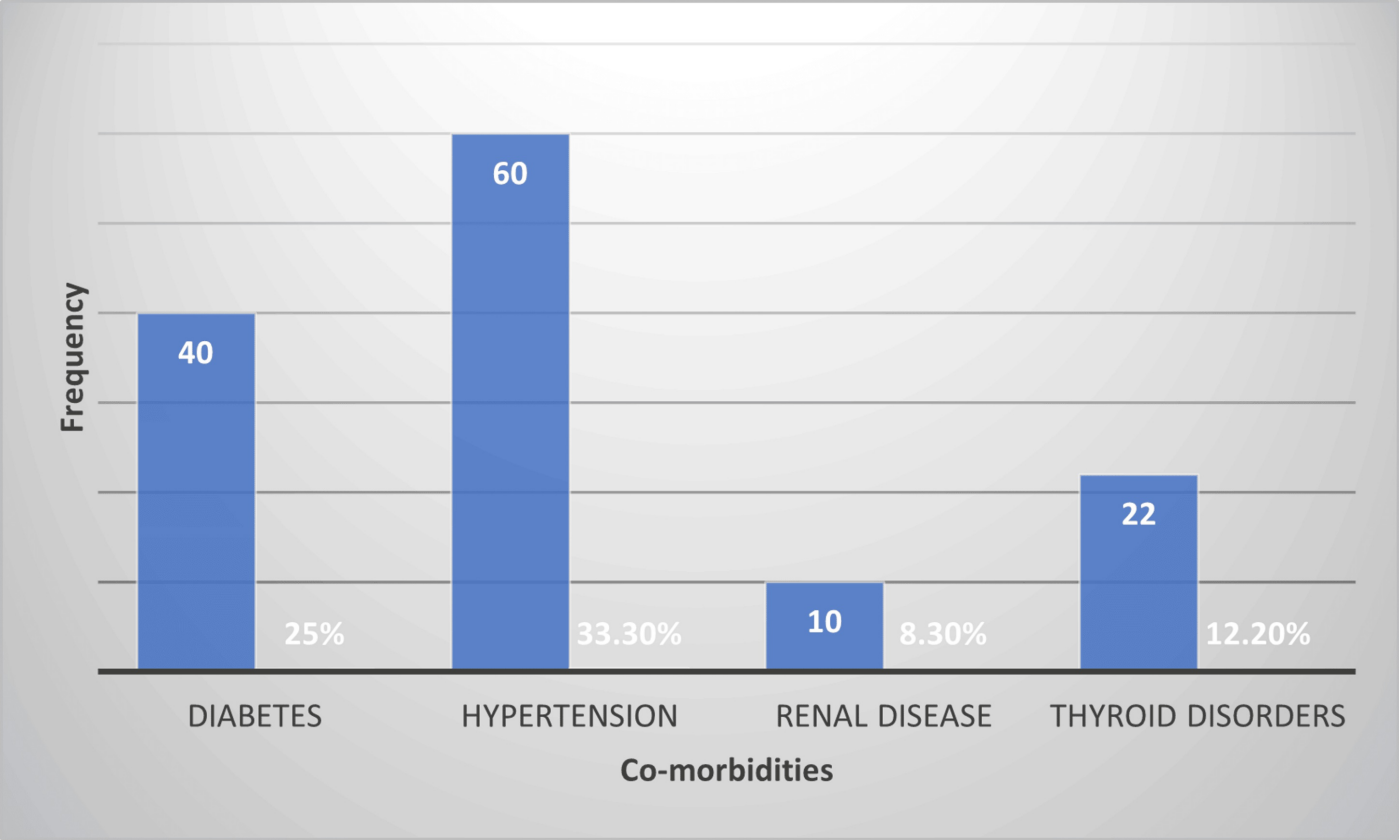
Prevalence of comorbidities in the study population.

Table 1 summarizes the age distribution of the 180 study participants. The age group of 46-60 years comprised the largest proportion of patients at 70 (38.9%), followed by 52 (28.9%) in the 31-45-year age group, 30 (16.7%) in the 18-30-year age group, and 28 (15.5%) who were older than 60 years. Figure 1 and Figure 2 display the gender distribution and comorbidity prevalence, presented as a pie chart and a column chart, respectively.

**Table 1:** Age distribution of study participants. Data are presented as frequency and percentage for categorical variables. No statistical tests were applied to this descriptive data.

Parameter	Frequency (n)	Percentage (%)
Age group (years)		
18–30	30	16.70%
31–45	52	28.90%
46–60	70	38.90%
>60	28	15.50%

Table 2 details the baseline hematological parameters of the study population. The mean hemoglobin level was 9.7 g/dL (±1.3), indicating mild-to-moderate anemia. The MCV was elevated at 104.7 fL (±9.8), which is consistent with macrocytic anemia. Reticulocyte count was low, with a mean of 0.7% (±0.3), while the white blood cell (WBC) count and platelet count were within normal limits, with means of 5,850 cells/mm³ (±1,480) and 225 × 10³/mm³ (±45), respectively. These results align with typical hematological findings in vitamin B12 deficiency, particularly affecting red blood cell parameters.

**Table 2:** Baseline hematological parameters of patients. Data are presented as mean ± standard deviation for continuous variables. No statistical tests were applied as this table describes baseline values. MCV: mean corpuscular volume; WBC: white blood cell

Hematological parameter	Mean ± SD
Hemoglobin (g/dL)	9.7 ± 1.3
MCV (fL)	104.7 ± 9.8
Reticulocyte count (%)	0.7 ± 0.3
WBC count (cells/mm³)	5,850 ± 1,480
Platelet count (×10³/mm³)	225 ± 45

**Figure 3:**
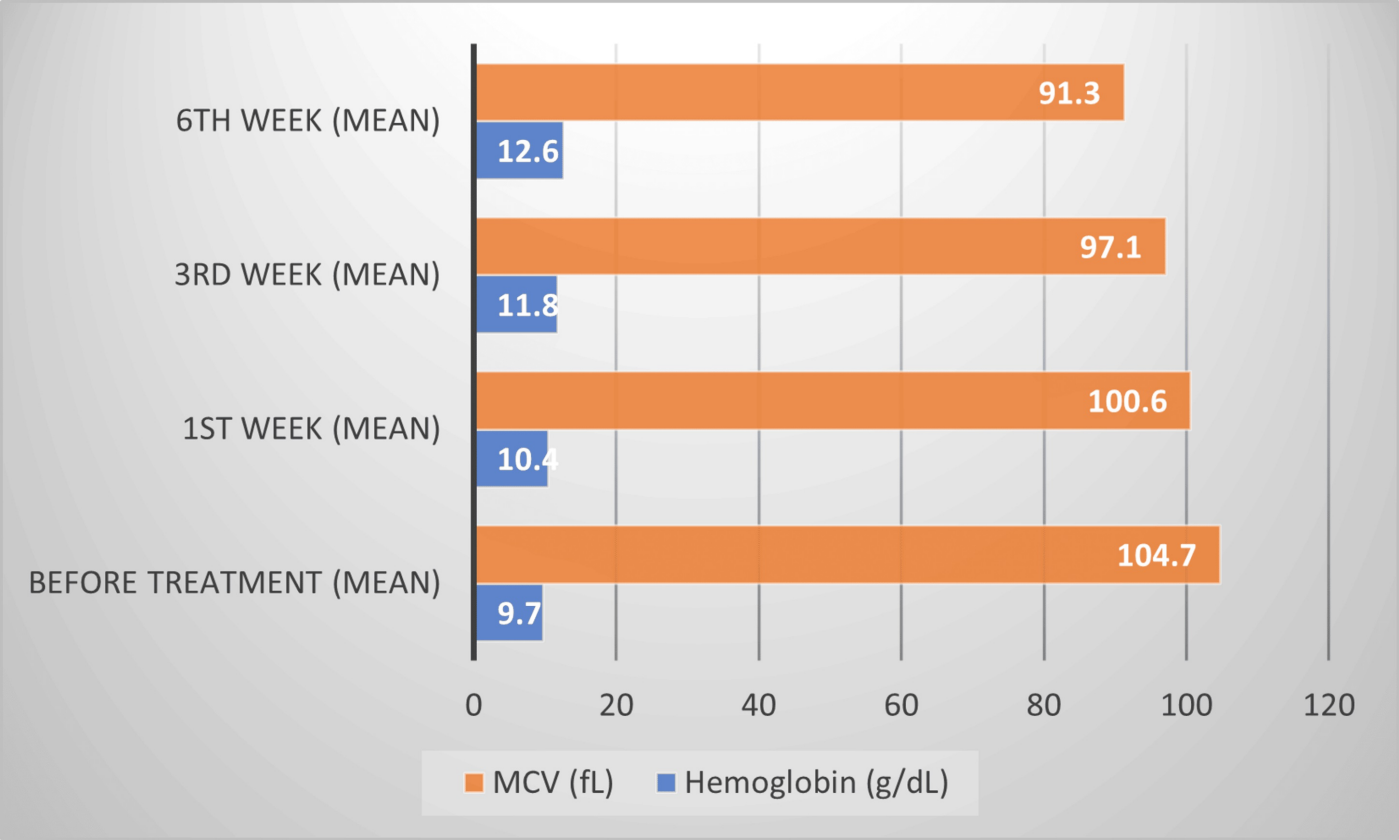
Changes in hemoglobin and MCV levels over time. MCV: mean corpuscular volume

Table 3 presents the hematological changes following vitamin B12 supplementation. Hemoglobin levels showed a significant increase from a baseline of 9.7 g/dL (±1.3) to 12.6 g/dL (±1.2) by the sixth week (p < 0.001, ANOVA). The MCV decreased progressively from 104.7 fL (±9.8) at baseline to 91.3 fL (±7.4) by the sixth week (p < 0.001), indicating correction of macrocytosis. The reticulocyte count increased from 0.7% (±0.3) to 2.1% (±0.5) (p < 0.001), reflecting improved red cell production. WBC and platelet counts also showed modest yet significant increases, with final counts of 7,150 cells/mm³ and 286 × 10³/mm³, respectively (p = 0.039 for WBC and p = 0.034 for platelets). These findings confirm that vitamin B12 supplementation effectively improves hematological parameters over time. Figure 3 illustrates the changes in hemoglobin and MCV levels over time, presented as a bar chart.

**Table 3:** Hematological changes after vitamin B12 supplementation. Data are presented as mean ± standard deviation. Statistical significance was assessed using one-way ANOVA to compare values at different time points. A p-value of less than 0.05 was considered statistically significant. ANOVA: analysis of variance; WBC: white blood cell

Parameter	Before treatment (mean ± SD)	First week (mean ± SD)	Third week (mean ± SD)	Sixth week (Mean ± SD)	P-value (ANOVA)
Reticulocyte count (%)	0.7 ± 0.3	1.1 ± 0.4	1.7 ± 0.5	2.1 ± 0.5	<0.001
WBC count (cells/mm³)	5,850 ± 1,480	6,250 ± 1,380	6,750 ± 1,320	7,150 ± 1,160	0.039
Platelet count (×10³/mm³)	225 ± 45	238 ± 53	268 ± 60	286 ± 64	0.034

Table 4 outlines the clinical symptoms associated with vitamin B12 deficiency. Fatigue was the most prevalent symptom, reported by 120 (66.7%) patients, followed by tingling or numbness by 98 (54.4%) patients. Light-headedness was reported by 82 (45.6%) patients, gastrointestinal symptoms by 48 (26.7%), and rapid heartbeat by 63 (35%). These results highlight the range of symptoms associated with vitamin B12 deficiency, with fatigue and neurological symptoms being particularly common.

**Table 4:** Clinical symptoms of vitamin B12 deficiency. Data are presented as frequency and percentage for categorical variables. No statistical tests were applied to this descriptive data.

Symptom	Frequency (n)	Percentage (%)
Fatigue	120	66.70%
Light-headedness	82	45.60%
Rapid heartbeat	63	35%
Tingling or numbness	98	54.40%
Gastrointestinal symptoms	48	26.70%

The results of Pearson’s correlation analysis between hemoglobin levels and serum vitamin B12 concentrations showed a significant positive correlation (r = 0.75, p < 0.001), indicating that lower levels of vitamin B12 are strongly associated with lower hemoglobin concentrations. This relationship underscores the direct impact of vitamin B12 deficiency on hemoglobin production and the development of anemia.

**Figure 4:**
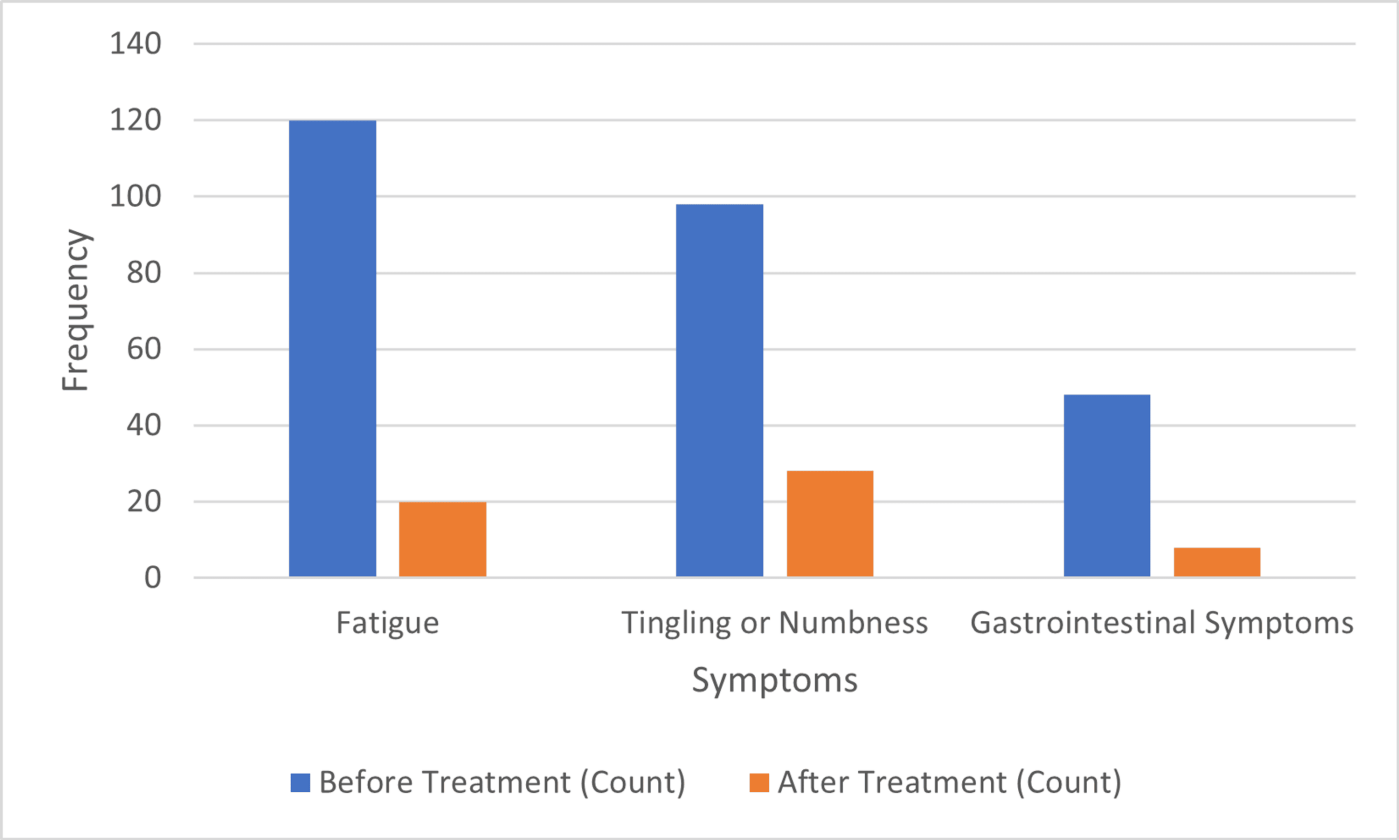
Clinical symptoms before and after vitamin B12 supplementation.

Table 5 shows the clinical improvements in symptoms after vitamin B12 supplementation. Fatigue, initially reported by 120 (66.7%) patients, decreased markedly to 20 (11.1%) after treatment (p < 0.001). Tingling or numbness also significantly declined, from 98 (54.4%) to 28 (15.6%) (p < 0.001). Gastrointestinal symptoms were similarly reduced, from 48 (26.7%) to 8 (4.4%) (p < 0.001). These results demonstrate that vitamin B12 supplementation effectively alleviates both neurological and gastrointestinal symptoms associated with deficiency. Figure 4 displays the clinical symptoms before and after vitamin B12 supplementation, presented as a column chart.

**Table 5:** Clinical improvement after vitamin B12 supplementation. Data are presented as frequency and percentage for categorical variables. A chi-square test was used to compare the prevalence of symptoms before and after treatment. A p-value of less than 0.05 was considered statistically significant.

Symptom	Before treatment, n (%)	After treatment, n (%)	P-value
Fatigue	120 (66.7%)	20 (11.1%)	<0.001
Tingling or numbness	98 (54.4%)	28 (15.6%)	<0.001
Gastrointestinal symptoms	48 (26.7%)	8 (4.4%)	<0.001

## Discussion

Vitamin B12 deficiency is one of the widest spectrums of clinical and hematological features and can be quite challenging to diagnose. The study fits well with what is already known about the demographic, clinical, and hematological profiles of patients with this condition, thereby giving a profound understanding of the therapeutic response after B12 supplementation. The research revealed that most study participants were middle-aged and older adults and had a higher prevalence among males. This finding aligns with other reports from South Asian countries in which vitamin B12 deficiency was common in those aged more than 45 years. Moreover, it has been reported that most patients suffer from comorbid conditions such as hypertension and diabetes [9,10]. Male predominance may be related to lifestyle and dietary factors, but the etiology is not well defined. This condition in India and Nepal has other significant risk factors, such as vegetarianism and a low intake of cobalamin-rich foods reported in previous studies [9,10].

The baseline hematological profile from this study presented macrocytic anemia as marked by increased MCV and decreased hemoglobin levels. However, it must be remembered that MCV is not always a sensitive marker when ruling out vitamin B12 deficiency as an isolated finding. According to Bhatia et al., MCV can be very misleading in cases of concurrent iron deficiency, where the macrocytic picture may get masked [11]. This finding is consistent with what we have observed in our study, whereby most patients had an extreme deficiency of vitamin B12 while still having normal MCV values. The hematological changes noted in this study are concordant with many previous studies [12-15]. Bhole et al. and Suthar et al. pointed out the wide spectrum of hematological abnormalities in vitamin B12 deficiency, which varies from normal hematological indices to overt megaloblastic changes [12,13]. The presence of macro-ovalocytes and hypersegmented neutrophils in the peripheral smear of affected patients confirms the diagnosis of megaloblastic anemia in advanced cases, as demonstrated in our study. Similar findings were noted in a study by Sakhare et al., where 80% of patients with vitamin B12 deficiency showed these classic features [16].

Clinically, the most common presenting symptom in our study was fatigue, followed by neurological complaints such as tingling or numbness in the extremities. These neurological manifestations are a hallmark of vitamin B12 deficiency and tend to appear in more advanced stages. Pandya et al. also reported similar neurological findings in their cohort of patients with megaloblastic anemia, highlighting the importance of early recognition and treatment to prevent irreversible damage [14]. Vitamin B12 deficiency also increases homocysteine levels, elevating cardiovascular risk. This interaction between vitamin B12 and homocysteine has been extensively studied, and Premkumar et al. noted that addressing B12 deficiency not only corrects anemia but also reduces homocysteine levels, thereby lowering the risk of thrombotic events [15]. While our study did not directly assess homocysteine levels, it is plausible that timely intervention with B12 supplementation mitigates this cardiovascular risk. The hematological response of vitamin B12 supplementation was impressive in this study. There was a striking increase in hemoglobin levels, and MCV values decreased by the sixth week. Reticulocyte counts also showed a highly significant increase, indicating recovery of bone marrow activity. This corresponds to the observations of other studies, which saw a similar hematologic benefit after B12 supplementation [16-19]. This further solidifies the efficacy of vitamin B12 therapy in the reversal of both clinical and hematological expressions of the deficiency.

Research conducted in different parts of India, including Maharashtra, has documented similar therapeutic responses; most patients experienced significant improvement in their symptoms and laboratory values after receiving vitamin B12 injections [17,20]. Srikanth et al. also documented the rapid reversal of neurological symptoms with early treatment, suggesting that the key aspect of treatment in such patients is early diagnosis and management. In this study, most patients were relieved of their presenting symptoms, especially those who presented with symptoms of fatigue and neurological complaints. This study’s well-established positive correlation between serum vitamin B12 levels and hemoglobin concentrations is consistent with previous reports. A similar correlation was documented by Aher et al., who established that serum vitamin B12 level is an effective indicator of the severity of anemia in affected patients [20]. Our significant correlation findings (r = 0.75 and p < 0.001) further endorse the current practice of using serum B12 levels for clinical assessment of patients suffering from anemia.

Strengths

One of the primary strengths of this study is its comprehensive approach to evaluating both the clinical and hematological manifestations of vitamin B12 deficiency in a well-defined population. The inclusion of patients with comorbidities such as hypertension and diabetes adds to the real-world applicability of the findings, as these conditions often coexist with anemia in clinical practice. Additionally, the use of a standardized treatment regimen and detailed follow-up assessments at multiple intervals ensure consistency in data collection and provide valuable insights into the short-term effects of vitamin B12 supplementation. The strong correlation observed between hemoglobin levels and serum vitamin B12 concentrations underscores the reliability of the diagnostic and therapeutic approaches employed in this study.

Limitations

The study has several limitations that should be considered when interpreting its findings. The small sample size may limit the generalizability of the results. As a single-center study, the findings may not apply to other regions or healthcare settings. Additionally, homocysteine levels, which are important for assessing cardiovascular risk in vitamin B12 deficiency, were not measured. The absence of folate and iron status assessment also limits the ability to rule out coexisting deficiencies, which could have influenced the hematological outcomes. Lastly, the six-week follow-up may not be sufficient to evaluate long-term therapeutic effects, particularly for neurological improvements and potential relapse, highlighting the need for extended follow-up in future studies.

## Conclusions

This study highlights the significant clinical and hematological impact of vitamin B12 deficiency, demonstrating its correlation with anemia and neurological symptoms. Vitamin B12 supplementation was shown to effectively correct anemia and alleviate neurological manifestations in deficient patients. These findings underscore the importance of early diagnosis and intervention, particularly in at-risk populations such as vegetarians and older adults. Given the widespread prevalence of B12 deficiency in the region, routine screening and supplementation are recommended. Further research is needed to assess the long-term benefits of B12 supplementation and its potential impact on reducing cardiovascular risk in deficient individuals.
